# Addressing Palliative Care Needs of COVID-19 Patients in New Orleans, LA: A Team-Based Reflective Analysis

**DOI:** 10.1089/pmr.2020.0057

**Published:** 2020-07-22

**Authors:** Rebecca V. Burke, Robin Rome, Kelly Constanza, Malaika Amedee, Charles Santos, Alexandra Leigh

**Affiliations:** ^1^ Section of Geriatrics and Extended Care, Southeast Louisiana Veterans Health Care System (SLVHCS), New Orleans, Louisiana, USA.; ^2^ Department of Internal Medicine, Tulane School of Medicine, New Orleans, Louisiana, USA.

**Keywords:** COVID-19, intensive care unit/intensive care unit issues in palliative care, palliative social work, palliative team

## Abstract

***Background:*** New Orleans, Louisiana served as a central location for a surge of novel coronavirus cases during the months of March 2020 to May 2020. To provide guidance to palliative care teams naive to the palliative care demand associated with a surge of coronavirus cases, we document our protocol to best optimize palliative care resources. This report aims to present this information and reflect upon what was most beneficial/least beneficial to serve as a roadmap for palliative teams facing this pandemic.

***Objective:*** To pilot a team-based structured protocol to categorize severity of COVID-19 intensive care unit (ICU) admissions and subsequently collaborate with the palliative interdisciplinary team to assess physical, spiritual, and psychosocial needs.

***Design:*** New ICU consults were categorized into color-coded clinical severity “pots” during daily ICU interdisciplinary rounds. Clinical decision making and communication with patient/next of kin were based on “pot” classification.

***Settings/Subjects:*** Palliative medicine consults were placed on all COVID-19 positive patients admitted to the ICU between March 29, 2020, and May 1, 2020.

***Measurements:*** A retrospective chart review was performed to analyze the effect of palliative care consultation on completion of goals-of-care conversations and the life-sustaining treatment (LST) document, an advance directive form specific to the Veterans Affairs hospital system between March 29, 2020 and May 1, 2020.

***Results:*** Of the palliative consults evaluated by a palliative provider, 74% resulted in completion of a LST document, 58% resulted in video contact with family members, and 100% incorporated a goals-of-care discussion.

***Conclusions:*** We found that standardizing palliative care consultation on all COVID-19 positive ICU admissions subjectively alleviated the burden on ICU providers and staff in the midst of a crisis, resulted in increased documentation of patient goals-of-care preferences/LSTs, facilitated clinical updates to family members, and better distributed clinical burden among palliative team members.

## Introduction

Communicating the complexity of medical illness to patients and families transparently and compassionately is a central duty of an inpatient palliative medicine team. Nonpalliative specialists have cited multiple challenges to servicing the palliative care needs of patients including lack of confidence or expertise, uncertainty with prognostication, fear of saying the wrong thing, and reluctance on the patient or family's part to talk about a worsening prognosis.^1–4^ Palliative medicine specialists strive to create a shared decision-making model with families that acknowledges the medical complexity and emotional difficulty of the situation, especially in the intensive care unit (ICU) setting.^5^

The palliative medicine team at Southeast Louisiana Veterans Health Care System (SLVHCS) was tasked with developing a model to provide comprehensive patient-centered care to ICU patients and their families. SLVHCS is a medical center located in New Orleans, Louisiana, providing health care to veterans throughout 23 parishes in Southeast Louisiana.^6^ SLVHCS identified the first COVID-19 positive patient in the state of Louisiana, and shortly thereafter cared for a surge of COVID-19 patients. During the month of March 2020, New Orleans, Louisiana had the highest growth rate per capita of new COVID-19 cases in the world.^7^ Our team-based model of care aimed to address the rapidly changing needs of these patients while preserving personal protective equipment (PPE), promoting staff well-being, and offloading work from front-line providers.

## Methods

The first step of incorporating the palliative team into the ICU surge plan of care involved attending daily ICU interdisciplinary rounds. Our team, previously working five days a week, transitioned to a seven-day work week including at least one provider daily. Key disciplines engaged in table rounds included the managing ICU team, nutrition, respiratory therapy, pharmacy, nursing, and social work. All COVID-19 positive ICU patients received a palliative consult.

**FIG. 1.**
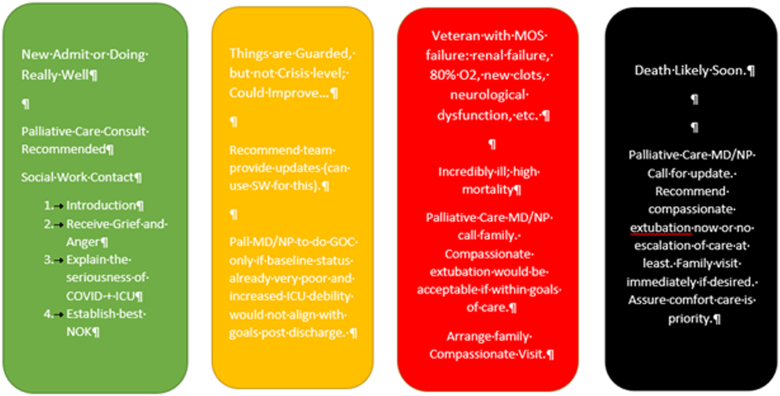
COVID-19 palliative care pots.

Each ICU COVID-19 patient was categorized daily during rounds into color-coded “palliative care pots” based on severity of illness (Fig. 1); designation was determined by the ICU fellow and attending. These pots allowed ICU, palliative, nursing, and other ICU team members to quickly grasp how the patient was doing medically.

New admissions to the ICU, stable, or recovering patients were designated to the “green pot.” The palliative social worker (SW) served as the primary consultant for “green pot” patients. Most green patients were on high flow nasal cannula, bi-pap, or were recently extubated. The SW would make phone contact with the patient (when possible directly to the hospital room) and/or next of kin (NOK). The SW would explain/assess points listed in Table 1 (all “green pot” patients). This work could all be done remotely.

**Table 1.** Palliative Care Social Work Phone Contact

Assessment	Prompt
Explain role of the palliative care team in the ICU during the COVID-19 pandemic. Receive grief/anger and other emotions from the NOK.	The palliative care team has been consulted by your primary ICU medical team. We focus specifically on symptom management (physical and psychological), supportive services, and advance care planning. The palliative care team adds an extra layer of support for you because COVID-19 is a serious and unpredictable illness. Although your medical team will provide updates, the palliative care team, including physicians, a nurse practitioner, SW, and chaplain are now also available to provide information and support.
Assess prior level of functioning and living arrangement.	Tell me about your (the veteran's) level of functioning before being admitted to the hospital.
	Were you independent at home?
	Do you require assistance with ADL/IADLs? If so, what type of assistance is received?
Identify health care NOK/legal surrogate. Completion of a health care power of attorney when patient able.	Have you completed a health care power of attorney or have you identified a person you would want to make health care decisions if you were not able?
Assess social support system (family/friends/employment). Assess availability of assistance at home.	Tell me about the type of support you have at home.
	Do you live alone?
	Do you have someone who can assist you at time of discharge if this is needed or someone you can move in with?
Assess patient's personality, hobbies, activities enjoyed. Share information with bedside nurses.	Tell me about hobbies and activities you enjoy.
	How would you describe your personality?”
Assess patient's spiritual or religious needs. For those seeking spiritual support, request a chaplain consult.	Do you have a spiritual preference or practice any religion? If yes, would you like a chaplain visit for prayer support?
Assess preferences (TV channels, glasses, hearing limitations). Share information with bedside nurses.	What channels do you enjoy watching on TV?
	Do you wear glasses, hearing aides, or dentures?
	What brings you joy?

ADL, activities of daily living; IADL, instrumental activities of daily living; ICU, intensive care unit; NOK, next of kin; SW, social worker.

“Yellow pot” patients were in serious condition, but not critical. Typically, these patients were ventilated and were weaning poorly from the ventilator, suffered from dysfunction of at least one organ (liver, kidney, or heart) besides the lungs, or were becoming more altered. Prior “green” patients who became “yellow” were transitioned to the palliative nurse practitioner (NP) or medical doctor (MD/DO) as the team lead, although social work team members might stay on for daily contact with family. Our palliative providers consisted of two palliative MDs and one palliative NP; this limitation in providers is what prompted us to delegate “green pots” to SW only. Family discussions focused on sharing medical status, discussing the prolonged nature of serious COVID-19 infection, and alerting to the potential for a poor outcome. Goals of care were broached, especially if prior functional status was poor. Yellow patients received frequent video visits with family.

The “red pot” was reserved for patients in multisystem organ failure not responding to therapy with worsening trajectory. These patients had a high likelihood of succumbing to the complications of COVID-19. The palliative care NP/MD was the lead for these patients. Prior visits with family had been electronic; brief in-person visits with appropriate PPE were arranged if family felt this might help their loved one recover and goals remained aggressive, or if the family wanted to say goodbye and were considering compassionate extubation.

A “black pot” was reserved for the most ill patients; death was imminent and expected at any time, even if goals of care were aggressive. The palliative care NP/MD was the lead for “black pot” patients. For patients in the “black pot,” a comfort measures only approach, including a compassionate extubation, or no further escalation of care was strongly recommended. Even when goals of care remained aggressive, family visits were offered to assure goodbyes could be said.

The daily schedule of our palliative care provider consisted of joining interdisciplinary ICU tabletop rounds in person, followed by rounding on the patients, assessing them through the glass doors, to limit PPE utilization and exposure, assuring that patients appeared comfortable. The palliative provider supported the ICU team with updates to family members for the more complicated patients in the yellow, red, or black pots. The palliative SW and primary ICU team provided clinical updates to green pot patients. The providers engaged in goals-of-care conversations and coordinated compassionate visits for family members whose loved ones were terminally ill with COVID-19. When patient's goals transitioned to comfort, compassionate extubation and comfort medication orders were entered to remove this workload from the ICU team. To preserve PPE, provider encounters inside COVID-19 patient rooms were minimal, mostly reserved for family video visits.

Besides providing brief medical updates and support to families of COVID-19 positive patients, the palliative care SW was responsible for identifying the bereavement needs for the families of the COVID-19 positive deceased patients. During the condolence call, the palliative care SW would provide education and normalization of the grief process. Information provided included community resources, financial resources, funeral home providers with cost, Veterans Affairs benefits information contact numbers, and obtaining the patient's personal effects. Collaboration with decedent affairs on specific issues was often needed. A facility psychologist was offered to patients and patient families wanting more support.

## Results

From a retrospective chart review of the 22 ICU consults placed during March 31, 2020 to May 1, 2020, 19 were palliative provider led (3 were SW phone visits only). We found that palliative provider consultation resulted in 74% (*n* = 14) completion rate of the life-sustaining treatments (LSTs) document, a Veterans Affairs specific advance directive form. Palliative providers completed video visits in 58% (*n* = 11) of consults and engaged in goals-of-care conversations in 100% (*n* = 19) of consults seen in person. This study primarily focused on ensuring code status was documented in the LST, although some LSTs documented more specific desires. Besides clarifying code status, the LST document allows veterans to state whether they would want ICU readmission, ventilation, blood products, dialysis, artificial nutrition or hydration, and their general wishes for their care (i.e., to live longer, to be cared for by loved ones, and to be comfortable). Further review of LST documentation among palliative consults indicated that 32% (*n* = 6) resulted in documentation of a new LST document, 26% (*n* = 5) resulted in confirmation/review of pre-existing LST (ensuring wishes were consistent), and 16% (*n* = 3) resulted in complete change in code status from pre-existing LST document.

## Discussion

We believe the team-based approach decreased the facility burden of caring for seriously ill COVID-19 patients through automating a palliative care consult, creating a quick communication strategy, and clearly delineating when to involve specific team members. Families contacted by palliative SWs expressed gratitude for the opportunity to ask questions freely and receive education on the medical status of their loved ones. Although this sample size was relatively small and collected over a short time period (about one month), this time period directly correlated with the surge of COVID-19 cases occurring in New Orleans, LA. COVID-19 positive cases started to downtrend after May 1, 2020, in the SLVHCS ICU. In addition, it is important to keep in mind that this series of patients required more time than a typical palliative care consult, as extensive coordination of care with family members through video rather than in person was deliberately arranged to preserve PPE and prevent exposure risks to families.

From our experience, expecting other doctors to “be” palliative care doctors during a crisis, or reserve time for self-education, was not feasible. We tried this approach and found that shared educational material was discarded, and family communication was lacking. Before integrating into ICU rounds, we trialed phone consults only. There simply was no time for the teams to contact us; we needed to be on site. Categorizing each patient to their respective “pot” ensured a consistent message was shared between teams. Before using the “pots,” it would often take extra time for the ICU and palliative care teams to fully understand how the patient medically was doing.

We found it most helpful to offload critical palliative needs by way of facilitating family meetings (through video, phone, or in person), confirming updates to NOK, walking ICU teams through comfort care or compassionate extubation orders, and supporting the emotional well-being of our medical peers. Lack of emotional support is a contributor to physician burnout, and although subjective, we felt that our physical presence in ICU rounds alleviated the psychological toll on nonpalliative clinicians tasked with frequently providing bad news.^9^

We admit early mistakes were made in our approach. Before frequent video visits becoming protocol, we found phone discussions with families about illness severity and code status talks challenging. It was not effective to give bad news as the first palliative contact with families; stunned families were unable to make surrogate decisions under those circumstances. After video visits were established, families were more aware of the severity of their loved ones' illness and willing to engage in these difficult discussions. The novelty of this virus made prognostication especially difficult, and although the “pots” streamlined communication, they were not prescriptive. Retrospectively, we learned that goals of care were most critical, as some patients with multiorgan system failure and aggressive goals did survive. We strongly encourage palliative providers to honestly communicate the clinical uncertainty of the COVID-19 trajectory to families and patients; this is critical to provide transparent medical care and appropriate support and counseling.

Lastly, we recommend a combined on-site and remote protocol for supporting ICU teams and patients during a COVID-19 surge. Automatic palliative care consults allowed for early palliative care support by off-site SWs who communicated daily with on-site palliative care providers. Communication tools such as “palliative care pots” helped teams communicate patient severity more easily. Consistent video communication with family facilitated understanding of the trajectory of their loved one. This helped the palliative care team quickly establish and maintain rapport for effective goals-of-care discussions.

## Abbreviations Used

ADL: activities of daily living
IADL: instrumental activities of daily living
ICU: intensive care unit
LST: life-sustaining treatment
NOK: next of kin
NP: nurse practitioner
PPE: personal protective equipment
SLVHCS: Southeast Louisiana Veterans Health Care System
SW: social worker
